# Cortical NO interneurons: from embryogenesis to functions

**DOI:** 10.3389/fncir.2013.00105

**Published:** 2013-06-03

**Authors:** Ludovic Tricoire, Yoshiyuki Kubota, Bruno Cauli

**Affiliations:** ^1^CNRS UMR7102, Neurobiologie des Processus Adaptatifs, Université Pierre et Marie CurieParis, France; ^2^Division of Cerebral Circuitry, National Institute for Physiological SciencesOkazaki, Japan

Neuronal processing and physiology of cortical circuits rely on a delicate interplay between glutamatergic excitatory neurons and GABAergic inhibitory interneurons in a spatially, temporally, and cell-type specific manner. Understanding these processes is further complicated by the large diversity characterizing the cerebral cortex (Ascoli et al., 2008; DeFelipe et al., 2013). Although recent advances have significantly improved our knowledge of its neuronal types, the identity and the roles of several subpopulations of GABAergic interneurons remain elusive (Gentet, 2012). Presumably, because of their apparent paucity, their diversity, the highly labile nature of nitric oxide (NO) as well as its pleiotropic actions, the functional importance of NO-producing GABAergic interneurons is particularly enigmatic. In this e-book we present a collection of articles published in *Frontiers in Neural Circuits* in response to the “Special topic” entitled “Cortical NO interneurons: from embryogenesis to functions.” Although it is practically impossible to address all aspects of NO interneurons, this special issue organized in four sections covers different facets of neocortical and hippocampal NO interneurons: from their diversity and embryonic origins to their functions in the cortical circuits and in neurovascular coupling.

Studying the functional roles of NO-producing cortical interneurons has been challenging. NO being a highly diffusible gas (Wood and Garthwaite, 1994) the identification and characterization of neurons producing it essentially rely on the direct or indirect detection of its synthesizing enzyme; the neuronal nitric oxide synthase isoform (nNOS). NO cortical interneurons have been historically classified as type I and type II on the basis of the size of their soma and by the intensity of their nNOS staining (Estrada and DeFelipe, 1998). Type I neurons, corresponding to heavily stained neurons with a large soma, have been studied at the molecular level but since they correspond to the rarest population of cortical interneurons (Kubota et al., 1994) their electrophysiological and pharmacological properties are poorly documented (Karagiannis et al., 2009; Kubota et al., 2011). In contrast type II neurons are much more numerous, but mainly for technical reasons inherent to the dimness of their staining, their properties and even their existence in the rodents has been questioned (Yan and Garey, 1997).

This issue was revaluated in the whisker-to-barrel cortex by using NADPH-diaphorase histochemistry (Nogueira-Campos et al., 2012) revealing that type I neurons were enriched in deep layers whereas type II NO interneurons were particularly dense in superficial layers. Consistently, using a combination of patch-clamp recordings, single cell RT-PCR and immunocytochemistry (Perrenoud et al., 2012a) disclosed that type I neurons were predominantly located in deeper layers and largely co-expressed somatostatin while type II neurons were concentrated in layers II/III and VI and heterogeneous at the molecular level. In contrast with the neocortex, the type I / type II subdivision does not hold in the hippocampus (Tricoire and Vitalis, 2012). By using an elegant spectral analysis to improve recognition of immunofluorescently labeled nNOS and neuropeptide-Y (NPY) expressing neurons (Somogyi et al., 2012), found that most NO interneurons with soma in the *stratum radiatum* of the CA1 area exhibit characteristic properties of ivy cells and express NPY. In contrast to their counterparts in the *stratum pyramidale* (Fuentealba et al., 2008), *stratum radiatum* ivy cells display a different pattern of axonal and dendritic arborizations suggesting that these two subpopulations of hippocampal NO interneurons are involved in different microcircuits.

Concomitantly with improvements in nNOS detection and the advent of genetic tools allowing lineage analysis of interneuron precursors, the developmental temporal and spatial origins of hippocampal and cortical nNOS neurons have been clarified with three studies showing that hippocampal and cortical type I NO interneurons originate from the medial ganglionic eminence (Jaglin et al., 2012; Magno et al., 2012; Perrenoud et al., 2012a), confirming and extending original findings (Tricoire et al., 2010). In contrast type II interneurons exhibited multiple embryonic origins (Magno et al., 2012; Perrenoud et al., 2012a). These works show that nNOS interneurons in addition of existing as different flavor also exhibit several and underestimated embryonic origins (Tricoire and Vitalis, 2012). These findings stress the still ongoing debate about the relative influences between the genetic programs and the molecular cues during embryogenesis.

Another important question addressed in this special topic, is how NO interneurons are modulated by afferent signals and what their main neuronal targets are. Type I NO interneurons were found to express selectively the NK1 receptor whose activation by its natural agonist substance P induced their depolarization (Dittrich et al., 2012). Using the phototoxicity of a NO indicator, selective damaging of cortical NO interneurons uncovered their role in lateral inhibition on neighboring columns and in the spatiotemporal dynamics of cortical activity (Shlosberg et al., 2012). A comprehensive review of the peculiar place of NO interneurons in cortical microcircuits is concluding this section (Armstrong et al., 2012).

Although NO is a well-established vasodilator, the relevance of NO interneurons in neurovascular coupling, the tight relationship between neuronal activity and local blood perfusion, has been questioned for decades (Cauli and Hamel, 2010; Kilduff et al., 2011). Besides, NO is also interacting with numerous signaling pathways involved in this process. The particularly complex roles of NO interneurons in the control of blood perfusion is extensively reviewed by (Duchemin et al., 2012) as an introduction to this section. As a first step in the understanding of their neurovascular functions, the anatomical relationships of type I interneurons with blood vessels is described in the rat cortical gray matter (Nogueira-Campos et al., 2012) and monkey white matter (Rockland and Nayyar, 2012) confirming and extending previous findings (Cauli et al., 2004). Stimulation of 5HT3A receptors expressed by type II NO interneurons and other discrete subpopulations of interneurons (Perrenoud et al., 2012b) leads to both NO-mediated vasodilations and NPY-mediated vasoconstrictions. These observations indicate that the role of NO interneurons in neurovascular coupling is even more complex when considering their diversity. In summary this special issue provides new important findings and up-to-date reviews on NO interneurons diversity and physiology that will be useful for both established scientists in this field and new comers.
